# Carfilzomib in multiple myeloma: unraveling cardiac toxicities - from mechanisms to diagnosis and management

**DOI:** 10.3389/fphar.2025.1570017

**Published:** 2025-03-27

**Authors:** Yi Gao, Di Zhou, Xue Bai, Yunjie Wang, Chenchen Wang, Lintao Bi

**Affiliations:** ^1^ Department of Cardiology, China-Japan Union Hospital of Jilin University, Changchun, China; ^2^ Department of Hematology, China-Japan Union Hospital of Jilin University, Changchun, China

**Keywords:** carfilzomib, cardiac toxicities, multiple myeloma, proteasome inhibitor, cardiooncology

## Abstract

The survival rates of patients with hematological malignancies such as multiple myeloma have improved with advances in cancer treatment. However, the risk of cardiovascular disease associated with novel therapeutic agents, including proteasome inhibitors (PIs), is becoming increasingly evident. PIs act on proteasome peptidases, leading to cell cycle arrest or apoptosis. Carfilzomib (CFZ), an intravenously administered irreversible PI, exhibits pronounced cardiovascular toxicity that is characterized by heart failure, hypertension, arrhythmia, and ischemic heart disease (IHD). This review focuses on CFZ, details its applications in treating multiple myeloma, presents its potential mechanisms of cardiotoxicity and the incidence of cardiotoxic events, and provides recommendations for the evaluation and management of adverse cardiac events during the early treatment of patients with this drug.

## 1 Introduction

Multiple myeloma (MM) accounts for approximately 10% of hematologic malignancies. It is characterized by the clonal proliferation of malignant plasma cells in the bone marrow, leading to the production of monoclonal proteins (often referred to as paraproteins) that can cause various systemic complications including renal dysfunction, anemia, bone lesions, and immunodeficiency (Rajkumar, 2024). Numerous patients can benefit from novel treatments and recent advances in the treatment of MM, such as immunomodulatory drugs, proteasome inhibitors (PIs), and emerging targeted therapies including epigenetic modulators and humanized monoclonal antibodies, leading to substantially prolonged survival rates (Engelhardt et al., 2024).

Carfilzomib (CFZ), a second-generation PI functions by inhibiting the proteasome, exerts its therapeutic effects by selectively inhibiting the β2 and β5 subunits of the proteasome, more precisely targeting malignant plasma cells with remarkable precision. This selective inhibition enhances the targeting accuracy and reduces off-target effects (Besse et al., 2019). In contrast to CFZ, bortezomib inhibition of the β5 subunit is reversible, resulting in a lower incidence of cardiovascular adverse events (CVAEs) (0.6–4.1% vs. 7–27% for CFZ) (Georgiopoulos et al., 2023). This difference may be attributed to the shorter half-life and greater target selectivity of bortezomib. Another proteasomal inhibitor, ixazomib, is associated with an even lower risk of cardiotoxicity (1.3%) (Georgiopoulos et al., 2023), likely due to its oral administration route and lower cumulative dose. The irreversible inhibition of both the β2 and β5 subunits enhances the anti-myeloma properties of carfilzomib but also increases the risk of cardiac injury by the persistent inhibition of the ubiquitin-proteasome system in cardiomyocytes.

CFZ can significantly enhance the survival rates of patients with MM. The clinical applications of CFZ in MM have transformed the treatment landscape, offering an effective option, particularly for patients with relapsed/refractory multiple myeloma (RRMM). CFZ was initially approved by the U.S. Food and Drug Administration in 2012 for patients with RRMM. Despite its effectiveness, CFZ treatment is associated with numerous adverse effects, among which CVAEs are a serious concern. Cardiovascular side effects, including hypertension (12.2%∼16%)^(6–8)^, heart failure (4.1%∼6.4%)^(6–8),^ IHD (1.8%∼6%)^(6, 9, 10)^, and arrhythmias (2.4%∼7%)^(6, 9, 10)^, can significantly impact patients’ overall health and limit the therapeutic benefit of CFZ (Bishnoi et al., 2021). Given the increasing use of CFZ in clinical practice, understanding the mechanisms underlying its cardiotoxicity and its impact on treatment outcomes is critical. Despite the urgent need for clear recommendations, assessment of the PI-related cardiotoxicity burden in patients with MM is impeded by inadequate data. This inadequacy stems from significant heterogeneity in defining cardiotoxicity endpoints, the exclusion of patients with high cardiovascular risk from clinical trials, and varying approaches in identifying and managing PI-related CVAEs. Consequently, as advanced therapies continue to improve the prognostic outcomes in patients at different stages of plasma cell disorders, the management of chronic treatment-related adverse effects has emerged as an increasingly pertinent concern. This review explores the clinical applications of CFZ in treating MM, focusing on its cardiovascular toxicities, the mechanisms involved, and current research strategies to mitigate these effects.

## 2 Use of CFZ in MM

The initial clinical use of CFZ in patients with RRMM was in combination with other agents. In early trials, such as the ASPIRE study in 2015, the clinical effects of CFZ, lenalidomide, and dexamethasone (KRd), and the lenalidomide and dexamethasone (Rd) regimen in the treatment of RRMM were compared. Compared with the Rd regimen, the KRd regimen could significantly improve the deep remission rate of patients, delay their relapse progression, improve their quality of life, and prolong their overall survival (OS) (Stewart et al., 2015). The following ENDEAVOR trial, which compared CFZ-based regimens with bortezomib-based regimens, demonstrated that 54% of patients treated with CFZ plus dexamethasone (Kd) achieved ≥ very good partial response (VGPR). Moreover, the study revealed that patients treated with bortezomib plus dexamethasone (Vd) exhibited superior progression-free survival (PFS) and OS compared with those treated with CFZ plus dexamethasone (Dimopoulos et al., 2016). The results from these studies have established CFZ as a cornerstone of treatment for patients with RRMM, even those who have been previously exposed to other PIs.

In recent years, among the clinical studies related to CFZ, most studies primarily focus on the clinical treatment of RRMM. These include the ARROW study (Takezako et al., 2021), which compared the administration regimens of CFZ at 70 mg/m^2^ once a week and 27 mg/m^2^ twice a week; the IKEMA study (Moreau et al., 2021), which evaluated the differences in therapeutic efficacy between the Isa-Kd (Isa monoclonal antibody combined with CFZ and dexamethasone) and Kd regimens; the CANDOR study (Usmani et al., 2022), which compared the DKd and Kd regimens. All of these are phase III clinical trials that had a large patient population. The research outcomes have a high clinical application value and deserve close attention. The FORTE study (Gay et al., 2021) ATLAS study (Dytfeld et al., 2023), and MASTER study (Costa et al., 2023) are other clinical studies conducted in patients with NDMM.

MM exhibits significant heterogeneity and individual variability in prognosis. Specific chromosomal abnormalities such as del (17p), del (1p), t (14; 16), t (14; 20), and t (4; 14) are frequently associated with an unfavorable prognosis and classified as high-risk MM (Rees and Kumar, 2024). A retrospective study conducted in 2023 at the Memorial Sloan Kettering Cancer Center included 154 patients with NDMM and high-risk cytogenetics. Patients treated with CFZ, lenalidomide, and dexamethasone (KRd) achieved a significantly better response compared with those treated with bortezomib, lenalidomide, and dexamethasone (VRd). Specifically, the rate of achieving at least a ≥ VGPR was 80% in the KRd group versus 65% in the VRd group. Moreover, KRd provided a notable survival advantage for patients with high-risk MM, with a median PFS of 70.9 months compared with 41 months for the VRd group (P = 0.016) (Tan et al., 2023). CFZ-based regimens have been effectively used in these challenging patient populations, offering a potential treatment option for patients with limited alternatives (Kumar et al., 2023).

The remarkable clinical efficacy of CFZ has attracted considerable attention. The high proteasome specificity of CFZ and its irreversible binding to the proteasome subunits contribute to its potent antimyeloma effects while minimizing off-target effects, such as a marked reduction in adverse events for neuritis (Besse et al., 2019). With continuing research and the development of combination regimens, CFZ is expected to remain an integral component of the treatment landscape for MM. During the course of treatment, it is imperative to closely monitor the CFZ-associated adverse reactions, particularly cardiovascular toxicity, to ensure that patients derive optimal benefits from the therapy and have minimal harm.

## 3 Cardiovascular complications in MM

The median age of patients diagnosed with MM is approximately ≥65 years (Mathur et al., 2017). Concurrent cardiovascular conditions are prevalent in this patient population, thereby elevating the risk of adverse events associated with the treatment of MM. A retrospective cohort study that included 32,193 patients with MM from the USA found that nearly two-thirds had heart disease at baseline, and overall incidence of cardiovascular events was 71%–72% over the 6-year study period (Kistler et al., 2012).

MM is associated with several disease-related factors that contribute to increased cardiovascular risk, including renal impairment, anemia, hyperviscosity, thrombosis, and light-chain amyloidosis (AL) (Fontes Oliveira et al., 2021). Notably, 50% of patients with MM exhibit abnormal renal function at diagnosis (Mikhael et al., 2021), which is linked to a higher risk of cardiovascular complications (Deo et al., 2023; Grams et al., 2023). Anemia is present in 73% of patients at diagnosis and 97% of patients during the course of MM (Kyle et al., 2003). It is independently associated with an elevated risk of cardiovascular disease and can exacerbate heart failure and pre-existing myocardial ischemia (Gan et al., 2023). Immunoglobulin light chain AL, characterized by the extracellular deposition of β-pleated sheet amyloid, which is resistant to degradation, may develop sub-clinically in up to 38% of patients with MM and progress to clinically overt AL in 10%–15% over the disease course. Cardiac involvement may manifest as heart failure (Desikan et al., 1997; Vela-Ojeda et al., 2009; Wang et al., 2022).

Considering that patients with MM are already at an elevated risk of cardiovascular disease at the time of diagnosis, the early identification and management of patients who are at high risk for CVAEs is crucial. CFZ has demonstrated significant efficacy in treating patients with MM, particularly those with RRMM. However, concerns have been raised about its cardiovascular toxicity, which manifests as hypertension, heart failure, IHD, and arrhythmia, potentially impacting the overall therapeutic outcome.

## 4 Evidence of CFZ-induced cardiotoxicity

Cardiovascular toxicity represents a prevalent and concerning adverse effect inassociated with PI therapy, with evidence suggesting a potentially higher incidence inwith CFZ treatment compared to other PIs (Bhutani et al., 2020). The incidence of CFZ-associated CVAEs varies among studies (Table 2). The incidence of CVAEs was 18.1% in a meta-analysis of 24 clinical trials involving patients treated with CFZ (Waxman et al., 2018). Another meta-analysis included 29 prospective clinical studies and a total of 4,164 patients with MM, and the incidence of all-grade and high-grade CVAEs in the CFZ group was 8.68% and 4.92%, respectively. No significant differences in the incidences of CVAEs were observed between patients with NDMM and RRMM in subgroup analyses (Shah et al., 2018).

The trend of CFZ-related adverse events in the Asian population, particularly in patients in Japan, along with the timing and clinical studies of CVAEs after their onset, indicates that CVAEs occur soon after CFZ administration. Specifically, the median time to onset for heart failure, congestive heart failure, and acute heart failure was approximately 2 weeks after treatment commencement (Nakao et al., 2022).

Several risk factors are associated with CFZ-induced CVAEs, including patient age, history of MM, prior and concurrent treatments, and drug dosage. Specifically, patients >65 years, those with a history of previous treatment for MM, and those undergoing concurrent treatment for MM demonstrated a higher incidence of CVAEs. A CFZ dose of ≥45 mg/m^2^ was linked to a significantly higher rate of high-grade CVAEs compared with doses <45 mg/m (Kyle et al., 2003). A single-center, retrospective analysis of 161 patients with MM treated with CFZ found that patients with a history of hypertension had a high risk of cardiotoxicity, as did those with a history of smoking. Thus, these findings suggest hypertension and smoking history as significant risk factors for CFZ-related cardiotoxicity across different patient populations (Doran et al., 2023). In a meta-analysis of 815 patients who were treated with CFZ, advanced age (>75 years) and having a history of cardiovascular disease, obesity, and a twice-weekly CFZ administration schedule were significant risk factors associated with CVAEs (Bishnoi et al., 2021). Furthermore, considering the elevated prevalence of hypertension and heart failure within the African American population (Yancy, 2024), this demographic may exhibit increased susceptibility to CFZ-induced cardiac events. Though race fails as a surrogate for genetics (Feero et al., 2024), preclinical genetic testing combined with race-specific risk assessment can reasonably predict and potentially optimize individualized medication strategies. For example, the gene variant V142I is one of the possible markers of genomic disease (Yancy, 2024). While the findings may not be universally applicable to all patients with MM, clinicians should be made aware of the high-risk factors that have been identified and advised to exercise increased vigilance and monitoring when using CFZ to treat patients with these risk factors.

Heart failure is a serious cardiovascular complication associated with CFZ therapy. It can markedly impact patients’ tolerance to treatment and diminish their overall quality of life. In an analysis of 5,583 patients across 45 prospective trials involving CFZ, the incidences of full-grade heart failure, edema, and ischemia were reported to be 5.1%, 20.7%, and 4.6%, respectively. The rates were 3.2% and 2.7% for high-grade heart failure and edema, respectively (Latif et al., 2021). This cardiotoxicity is particularly concerning in patients having preexisting cardiovascular risk factors such as diabetes, obesity, or a history of cardiovascular disease (Bishnoi et al., 2021).

In a systematic review and meta-analysis, arrhythmic events including atrial fibrillation (AF), conduction disturbances, and ventricular and supraventricular arrhythmias were reported in 7% of patients treated with CFZ (Bishnoi et al., 2021). Limited data are available on the prevalence of specific cardiac arrhythmias other than AF. Arrhythmias can lead to adverse outcomes including increased hospitalization rates and prolonged therapy with antiarrhythmic agents. The risk of developing arrhythmias is higher in older patients, those with a history of cardiac arrhythmias, or patients prescribed concurrent medications that can affect the electrical conduction system of the heart (Bishnoi et al., 2021). *In vivo* studies in mice suggest the role of the immuno-proteasome subunit PSMB10 in the molecular mechanism of AF (Li et al., 2018); however, no study has directly reported the pathophysiological mechanisms underlying CFZ-induced arrhythmia.

Hypertension is the most frequently observed complication resulting from CFZ use. The ENDEAVOR study found the overall incidence of CFZ-related any-grade hypertension to be 13.2% and that of high-grade hypertension to be 5.3% (Latif et al., 2021). Similarly, the ASPIRE trial reported a higher incidence of hypertension in the KRd group than in the Rd group (4.3% vs 1.8%) (Stewart et al., 2015). A retrospective analysis utilizing the Surveillance, Epidemiology, and End Results-Medicare–linked database evaluated adverse cardiac events in 635 patients treated with CFZ and found that 22% of patients developed hypertension (Fakhri et al., 2020).

Notably, the aforementioned trials used to evaluate CVAEs frequently lacked standardized protocols and were not consistently validated by cardiology professionals. Cardiovascular events are known to result in dose reductions or treatment interruptions, ultimately leading to the suboptimal management of MM. Therefore, given the importance of maximizing treatment efficacy in patients with MM, it is crucial for clinicians to closely monitor their cardiovascular health throughout the course of CFZ therapy. Identifying high-risk patients, implementing preventive strategies, and effectively managing cardiotoxicity are key approaches to ensure the continued success of CFZ and minimizing its cardiovascular risks when treating MM.

## 5 Mechanisms of CFZ-related cardiotoxicity

**FIGURE 1:**
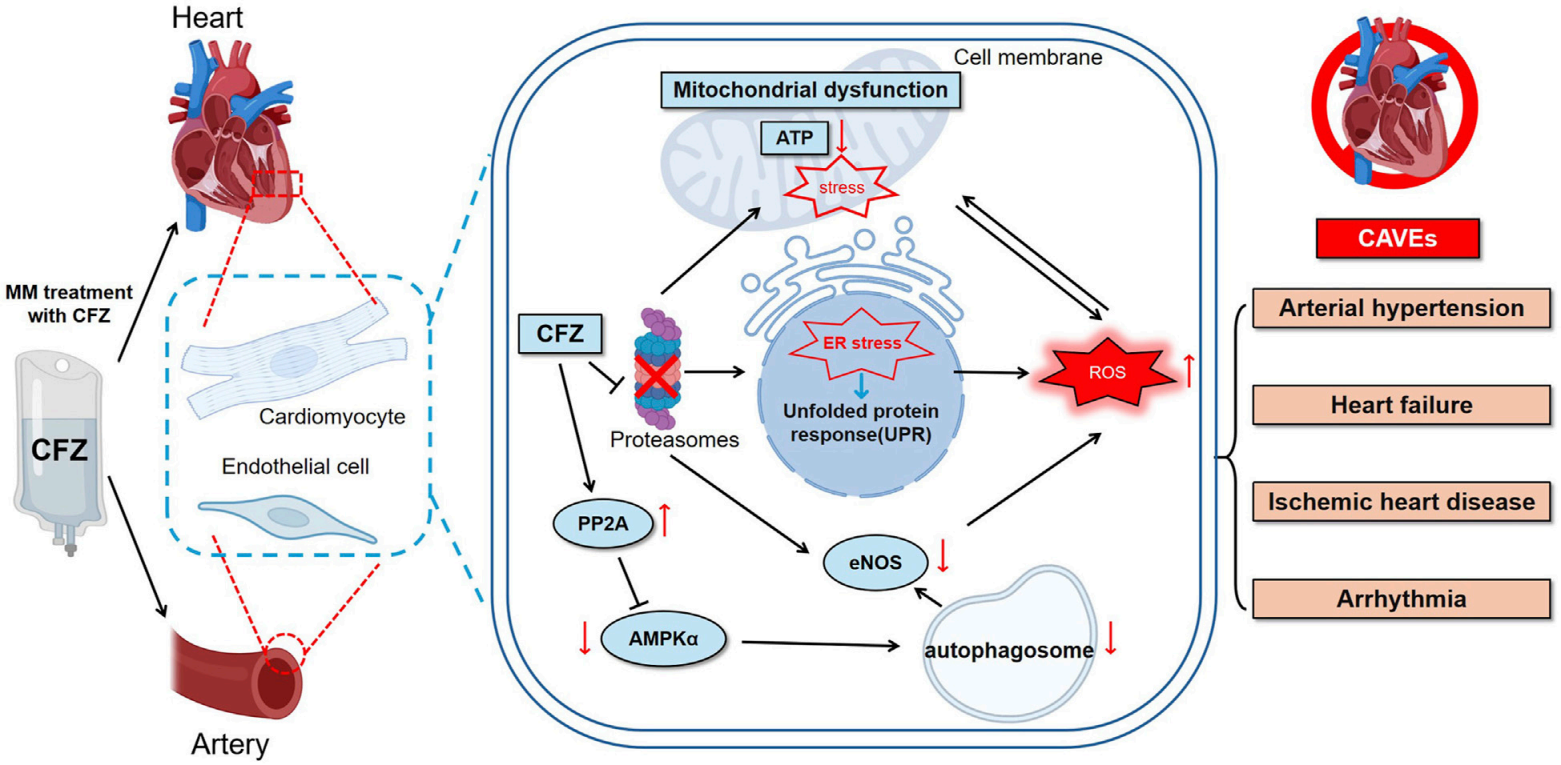
CFZ can trigger a diverse range of CVAE, such as hypertension, heart failure, ischemic heart disease, and arrhythmia. The underlying mechanisms may include the UPR, ER stress, ROS accumulation, and mitochondrial dysfunction. Arrows indicate the sequence of these events. CFZ = carfilzomib, eNOS = endothelial nitric oxide synthase, ER = endoplasmic reticulum, ROS = reactive oxygen species, UPR = unfolded protein response, CVAEs = cardiovascular adverse events, PP2A = protein phosphatase 2A.

The mechanisms underlying the cardiotoxicity of CFZ remain to be fully elucidated. However, these mechanisms may involve both direct myocardial injury and indirect effects mediated through changes in vascular function, blood pressure regulation, and the induction of inflammatory responses. A comprehensive understanding of PI-induced cardiotoxicity may facilitate the further development of preventive and therapeutic strategies (Figure 1) (Table 1).

**TABLE 1:** Experimental studies of CFZ effect on endothelial cells and cardiac cells.

Year	Experimental model	Dose of CFZ	Duration of PI	Results
2019 (Efentakis et al., 2019)	Mice	8 mg/kg	2 doses/every 48 h for 6 days	Cfz decreased left ventricular function through increased PP2A activity and inhibition of AMPKα and its downstream autophagic targets
2017 (Hasinoff et al., 2017)	Primary neonatal rat myocyte	0.1, 0.3, 1 and 3 μM	72 h	CFZ induced apoptosis, inhibited the chymotrypsin-like proteasomal activity of myocyte lysate in the low nanomolar concentration range and exhibited time-dependent inhibition kinetics
2024 (George et al., 2024)	C57BL/6 mice	8 mg/kg	a dose for 2 consecutive days	CFZ induced endoplasmic reticulum stress and inflammation
2017 (Imam et al., 2017)	Wistar albino male rats	4 mg/kg	six doses of CFZ	CFZ increased release of cardiac enzymes such as LDH, CK and CK-MB. CFZ significantly downregulated α-MHC mRNA expression while upregulated β-MHC and BNP mRNA expression. CFZ significantly upregulated NF-κB mRNA expression while downregulated p53 mRNA expression
2021 (Efentakis et al., 2021)	Aged mice	8 mg/kg	2 days	CFZ decreased proteasome activity, and increased myocardial oxidative stress. CFZ reduced AMPKα phosphorylation and increased bip expression, without increasing PP2A activity. CFZ increased PP2A activity, reduced AMPKα phosphorylation and increased bip and LC3B-depedendent autophagy
2024 (Hjazi et al., 2024)	HUVECs	0.05, 0.1, 0.2, and 0.5 µM	24 h	CFZ is unable to induce ER stress in confluent resting endothelial cells and can conversely attenuate the prothrombotic effects of TNFα on the endothelium
2023 (Dabour et al., 2023)	Endothelial cells (HUVECs and EA.hy926 cells)	0.05, 0.1, 0.2, 0.5, and 1 µM	24 h	Carfilzomib decreases the viability of endothelial cells by inducing apoptosis.CFZ increased the expression of ICAM-1 and VCAM-1. activated Akt and MAPK pathways, inhibited p70s6k pathway, and downregulated AMPK pathway
2025 (Dabour et al., 2025)	Endothelial cells (HUVECs and EA.hy926)	0.05, 0.1, 0.2, and 0.5 µM	24 h	CFZ induces ER stress and autophagy in endothelial cells
2021 (Forghani et al., 2021)	hiPSC-CMs	0.01μM∼10 μM	24 h, 48 h	CFZ treatment reduced mitochondrial membrane potential, ATP production, and mitochondrial oxidative respiration and increased mitochondrial oxidative stress. CFZ treatment impaired Ca2+ transients and reduced integrin‐mediated traction forces. CFZ treatment downregulated the expression of genes involved in extracellular matrices, integrin complex, and cardiac contraction, and upregulate stress responsive proteins including heat shock proteins
2023 (Jannuzzi et al., 2023)	H9C2 cardiomyocytes	10, 25, 50, 100, 250, 500 nM, and 1 μM CFZ	24 h	CFZ upregulated the ER stress protein (HSP90, HSP70, GRP94, and GRP78) levels, and reduced mitochondrial membrane potential and ATP production
2024 (Wesley et al., 2024)	C57BL/6J male mice	8 mg/kg	8 mg/kg ip on days 1, 2, 5 and 6	A steeper pressure-stiffness curve was observed for CFZ in normotensive and hypertensive mice. CFZ could induce systolic cardiac dysfunction in hypertensive mice
2017 (Chen-Scarabelli et al., 2017)	Isolated rabbit heart and aorta	10^–9^, 10^–8^ and 10^−7^ mol/L	——	CFZ increased coronary perfusion pressure, resting vasoconstricting tone and the spasmogenic effect of different agents.CFZ can impair vasodilation via an endothelium dependent mechanism

ATP, adenosine triphosphate, BNP = B-typeNatriureticPeptide, CK, creatine kinase; CFZ, carfilzomib; ER, endoplasmic reticulum; hiPSC-CMs, human induced pluripotent stem cell-derived cardiomyocytes; HSP, heat shock protein; HUVECs, human umbilical vein endothelial cells; LDH, lactate dehydrogenase; MHC, myosin heavy chain; NF-κB, nuclear factor kappa-B, PP2A = protein phosphatase 2A.

**TABLE 2:** Clinical trial data on CFZ-associated cardiotoxicity.

Author year	Drugs	Name	Sample	Efficacy	HF (All grade)	HF (Grade ≥3)	IHD (All grade)	IHD (Grade ≥3)	Thrombosis (All grade)	Thrombosis (Grade ≥3)	HTN (All grade)	HTN (Grade ≥3)	Arrhythmia (All grade)	Arrhythmia (Grade ≥3)
Facon et al. (2019)	KMP vs VMP	CLARIAN	955	MPFS: 22.3 m vs 22.1 m. ORR: 84.3% vs 78.8%. CRR: 25.9% vs 23.1%. MRD-negative rate: 15.7% vs 15.5%	51 (10.8%) vs 20 (4.3%)	39 (8.2%) vs 13 (2.8%)	14 (3%) vs 9 (1.9%)	10 (2.1%) vs 6 (1.3%)	——	——	——	——	——	——
Astarita et al. (2023)	Kd vs KRd	——	109	——	5 (10.6%) vs 2 (3.2%)	2 (4.3%) vs 2 (3.2%)	——	——	——	——	26 (55.3%) vs 22 (35.5%)	——	4 (8.5%)vs 5 (8.0%)	——
Kumar et al. (2020)	KRd vs. VRd	ENDURANCE	1,087	MPFS: 34.6 m vs 34.4 m	——	19 (4%)vs 6 (1%)	——	——	——	26 (5%)11 (2%)	——	22 (5%) vs 11 (2%)	——	——
Dimopoulos et al. (2016)	Kd vs. Vd	ENDEAVOR	929 RRMM	MPFS: 18.7 m vs months in 9.4 m	38 (8.2%) vs 11 (2.9%)	22 (5.2%) vs 6 (1.9%)	——	——	——	——	115 (25%) vs 40 (9%)	41 (9%) vs 12 (3%)	——	——
Takezako et al. (2021)	Once-weekly vs. twice-weekly CFZ	ARROW	40	MPFS: 14.8 m vs 9.7 m. ORR: 73.1% vs 57.1%	2 (7.7%) vs 2 (14.3%)	2 (7.7%) vs 1 (7.1%)	——	——	——	——	8 (30.8%) vs 5 (35.7%)	3 (11.5%) vs 2 (14.3%)	——	——
Stewart et al. (2015)	KRd vs. Rd	ASPIRE	792	MPFS: 26.3 m vs 17.6 m. OS: 73.3% vs 65.0%	25 (6.4%) vs 16 (4.1%)	15 (3.8%) vs 7 (1.8%)	23 (5.9%) vs 18 (4.6%)	13 (3.3%) vs 8 (2.1%)	——	——	56 (14.3%) vs 27 (6.9%)	17 (4.3%) vs 7 (1.8%)	——	——
Moreau et al. (2021)	IKd vs Kd	IKEMA	302	MPFS: not reached vs 19.15 m	13 (7%) vs 8 (7%)	7 (4%) vs 5 (4%)	8 (5%) vs 5 (4%)	2 (1%) vs 2 (2%)	27 (15%) vs 20 (16%)	7 (4%) vs 7 (6%)	65 (37%) vs 38 (31%)	36 (20%) vs 24 (20%)	——	——
Usmani et al. (2022)	DKd VS Kd	CANDOR	466	Median follow-up: 27.8 m vs 27.0 m. MPFS: 28.6 m vs 15.2 m	27 (10%) vs 17 (12%)	12 (5%) vs 13 (9%)	——	——	——	——	109 (35%) vs 46 (30%),	65 (21%) vs 23 (15%)	——	——
Gay et al. (2021)	induction and consolidation:KRd plus ASCT vs KRd12 vs KCd plus ASCT maintenance: KR vs R	FORTE	474	3-year PFS: 75% versus 65%	2 (1%) vs 0vs3 (2%) vs 3 (2%)vs0	0vs0 vs 1 (1%)vs3 (2%) vs0	1 (1%) vs 1 (1%) vs0vs2 (1%) vs 1 (1%)	1 (1%) vs 1 (1%) vs0vs2 (1%) vs 1 (1%)	11 (7%) vs 10 (6%) vs 4 (3%) vs 4 (2%) vs 1 (1%)	0 vs 0 vs 1 (1%) vs 0 vs 0	7 (5%) vs 18 (11%) vs 14 (9%) vs 24 (13%)vs0	3 (2%) vs 10 (16%) vs 4 (3%) vs 6 (3%)vs10	2 (2%) vs 5 (4%) vs 3 (2%) vs 4 (2%)vs0	1 (1%) vs 1 (1%) vs 0 vs 3 (2%)vs0
Dytfeld et al. (2023)	KRd vs R	ATLAS	180 NDMM	MPFS: 59.1 m vs 41.4 m	——	——	——	——	2 (2%) vs 3 (3%)	——	——	——	——	——
Hájek et al. (2017)	K vs Cd	FOCUS	315	Median OS: 10.2 m vs 10.0 m. ORR: 19.1% vs 11.4%	10 (7%) vs 2 (2%)	3 (2%) vs 1 (1%)	——	——	——	——	28 (18%)vs9 (6%)	5 (3%)vs0	——	——
Siegel et al. (2018)	KRd vs Rd	ASPIRE	792 RRMM	Median OS: 48.3 m vs 40.4 months for Rd	45 (11.4% vs 24 (6.2%)	17 (4.3% vs 8 (2.1%)	42 (10.7% vs 27 (6.9%)	15 (3.8% vs 9 (2.3%)	——	——	92 (23.5% vs 43 (11%)	25 (6.4% vs 9 (2.3%)	——	——
Jackson et al. (2021)	KRdc vs Tdc vs Rdc	——	1056 NDMM	3-year PFS of KRdc vs control: 64.5% vs 50.3%	5 (1%)vs0vs0	4 (0.8%)vs0vs0	——	——	48 (9.5%) vs 24 (9.4%) vs 15 (5.8%),	10 (2%)vs6 (2.4%)vs4 (1.6%)	3 (0.6%) vs 2 (0.8%) vs 1 (0.4%)	2 (0.4%) vs 0 vs 1 (0.4%)	——	——
Gregersen et al. (2022)	Kd vs observation		168	Median TTP: 25.1 vs 16.7 m	——	——	——	——	1 (1%)vs0	1 (1%)vs0	15 (18.3%) vs 3 (3.5%)	3 (4%) vs 1 (1%)	——	——
Yong et al. (2021)	KCd vs VCd	MUKfive	300	MPFS: 11.9 m vs 5.6 m. Median OS: 25.7 m vs 24.1 m	——	——	——	——	——	——	10 (5.1%) vs 2 (2.1%)	7 (3.6%)vs0	——	——

Cd = corticosteroids and cyclophosphamide, CFZ, carfilzomib; CRR, complete response rate; HF, heart failure; HTN, hypertension; IKD, isatuximab plus CFZ-dexamethasone, KD = CFZ, plus dexamethasone; KMP, carflzomib plus melphalan-prednisone, K = CFZ, monotherapy, KR = CFZ, plus lenalidomide, KRdc = CFZ, plus lenalidomide plus fdexamethasone plus cyclophosphamide; MRD, minimal residual disease; mPFS, median progression-free survival; ORR, overall response rate; OS, overall survival, R = lenalidomide, Rdc = lenalidomide plus dexamethasone-cyclophosphamide, Tdc = thalidomide plus dexamethasone-cyclophosphamide, TTP, time to progression; VMP, bortezomib plus melphalan-prednisone.

### 5.1 Direct myocardial injury

#### 5.1.1 Ubiquitin-proteasome system (UPS) dysregulation

CFZ, a second-generation PI, exerts direct cardiotoxicity through its impact on the ubiquitin-proteasome system (UPS). UPS is a primary pathway for protein degradation in cells, accounting for the degradation of >80% of cellular proteins (Dale et al., 2021). It plays a crucial role in degrading dysfunctional or unnecessary proteins and effectively maintain cellular homeostasis (Inobe and Matouschek, 2014). PIs can block proteasomal activity. Proteasome inhibition in MM cells results in the rapid accumulation of misfolded regulatory proteins in the endoplasmic reticulum (ER). This outcome subsequently triggers ER stress and the unfolded protein response (UPR), initiating a cascade of apoptotic events that culminate in the apoptosis of MM cells (Merin and Kelly, 2014; Tang et al., 2024; Moreau et al., 2012). Unlike other cell types, cardiomyocytes are nonproliferative cells characterized by higher proteasome activity and protein turnover rates that make them particularly susceptible to proteasome inhibition (Casula et al., 2009; Hedhli and Depre, 2010). Inhibition of proteasome-dependent protein turnover in cardiomyocytes can lead to protein imbalance, abnormal accumulation of ubiquitinated proteins, and formation of protein aggregates, resulting in cellular dysfunction, caspase-mediated apoptosis, and cell death (Guo et al., 2020; Hasinoff et al., 2017).

#### 5.1.2 Mitochondrial dysfunction

CFZ disrupts energy metabolism in cells by interfering with mitochondrial functions. The outcome is the accumulation of specific subunit proteins in the mitochondrial respiratory chain complex, potentially impeding the electron transport chain and reducing the efficiency of adenosine triphosphate (ATP) production by the mitochondria, thereby resulting in a decrease in cellular energy (Mendez et al., 2021). Using human induced pluripotent stem cell-derived cardiomyocytes as a model to investigate drug-induced cytotoxicity, studies have found that treatment with CFZ can reduce mitochondrial membrane potential, ATP production, and oxidative respiration in mitochondria, while simultaneously increasing oxidative stress in the mitochondria. These changes cause structural and functional alterations in cardiomyocytes, resulting in cardiotoxicity (Forghani et al., 2021; Jannuzzi et al., 2023). A multiomics integrative analysis revealed a significant downregulation of pyruvate and a concurrent upregulation of lactate dehydrogenase B in patients with CVAE after CFZ treatment. These findings suggest that the cardiotoxic effects of CFZ are likely related to mitochondrial dysfunction (Tantawy et al., 2021).

#### 5.1.3 Autophagy dysregulation

Enhanced protein phosphatase 2A (PP2A) activity and disruption of autophagy via the inhibition of AMPKα and its downstream autophagy-related targets can significantly contribute to CFZ-induced left ventricular (LV) dysfunction in mice (Papanagnou et al., 2018). CFZ can impair LV function by upregulating PP2A activity and suppressing AMPKα and its downstream autophagy pathways, suggesting autophagy disruption as a key mechanism underlying the cardiotoxicity of CFZ (Efentakis et al., 2019; Efentakis et al., 2024).

### 5.2 Vascular endothelial dysfunction

#### 5.2.1 eNOS/NO pathway impairment

Increasing evidence demonstrates the critical role of UPS in regulating the expression and activation of endothelial nitric oxide synthase (eNOS), endothelium-dependent contractile and vasodilator factors, and endothelial oxidative stress responses (Stangl et al., 2004; Stangl and Stangl, 2010). Given the pivotal role of UPS in modulating the functions of eNOS, it is logical to explore the potential of inhibiting the downstream effects of UPS on eNOS. While direct experimental evidence remains limited, it can be reasonably hypothesized that CFZ may inhibit the reduction in eNOS activity and nitric oxide (NO) production. NO plays a role in maintaining vasodilation and normal blood flow. Decreased NO production can result in vasoconstriction, increased blood pressure, and increased cardiac afterload (Maneesai et al., 2023; Bank et al., 1994).

#### 5.2.2 Thrombotic microangiopathy

Thrombotic microangiopathy developed in 5% of patients with MM treated with CFZ (Fotiou et al., 2020). It was characterized by endothelial damage that activated the coagulation cascade, consumption coagulopathy, and other factors that ultimately led to microangiopathic hemolytic anemia, platelet consumption, fibrin deposition, and small-vessel thrombosis (Bhutani et al., 2020). The effects of CFZ on the renal endothelium may implicated in the pathogenesis of these complications and may share a common pathophysiology with the cardiovascular effects of CFZ.

### 5.3 Systemic inflammatory responses

#### 5.3.1 Oxidative stress and ROS generation

*In vitro* studies with CFZ in the aortic smooth muscle cells of both mice and humans have demonstrated an increase in intracellular reactive oxygen species (ROS) production (Efentakis et al., 2020). Excessive ROS can damage biological macromolecules such as lipids, proteins, and nucleic acids in cardiomyocytes, leading to lipid peroxidation, disruptions of the structure and functions of cell membranes, and apoptosis or necrosis, thereby contributing to cardiovascular toxicity (Mongirdienė et al., 2022).

#### 5.3.2 Pro-inflammatory cytokine release

A patient with MM experienced fatal acute heart failure following CFZ administration. Autopsy revealed inflammatory cell infiltration between myocardial cells (Takakuwa et al., 2019). CFZ can activate oxidative stress and inflammatory responses, leading to the release of inflammatory mediators including tumor necrosis factor-α, interleukin (IL)-1β, and IL-6 (Alam et al., 2022). These factors exacerbate myocardial inflammation and promote myocardial fibrosis.

## 6 Strategies for prevention and management of CFZ-induced CVAEs

**FIGURE 2:**
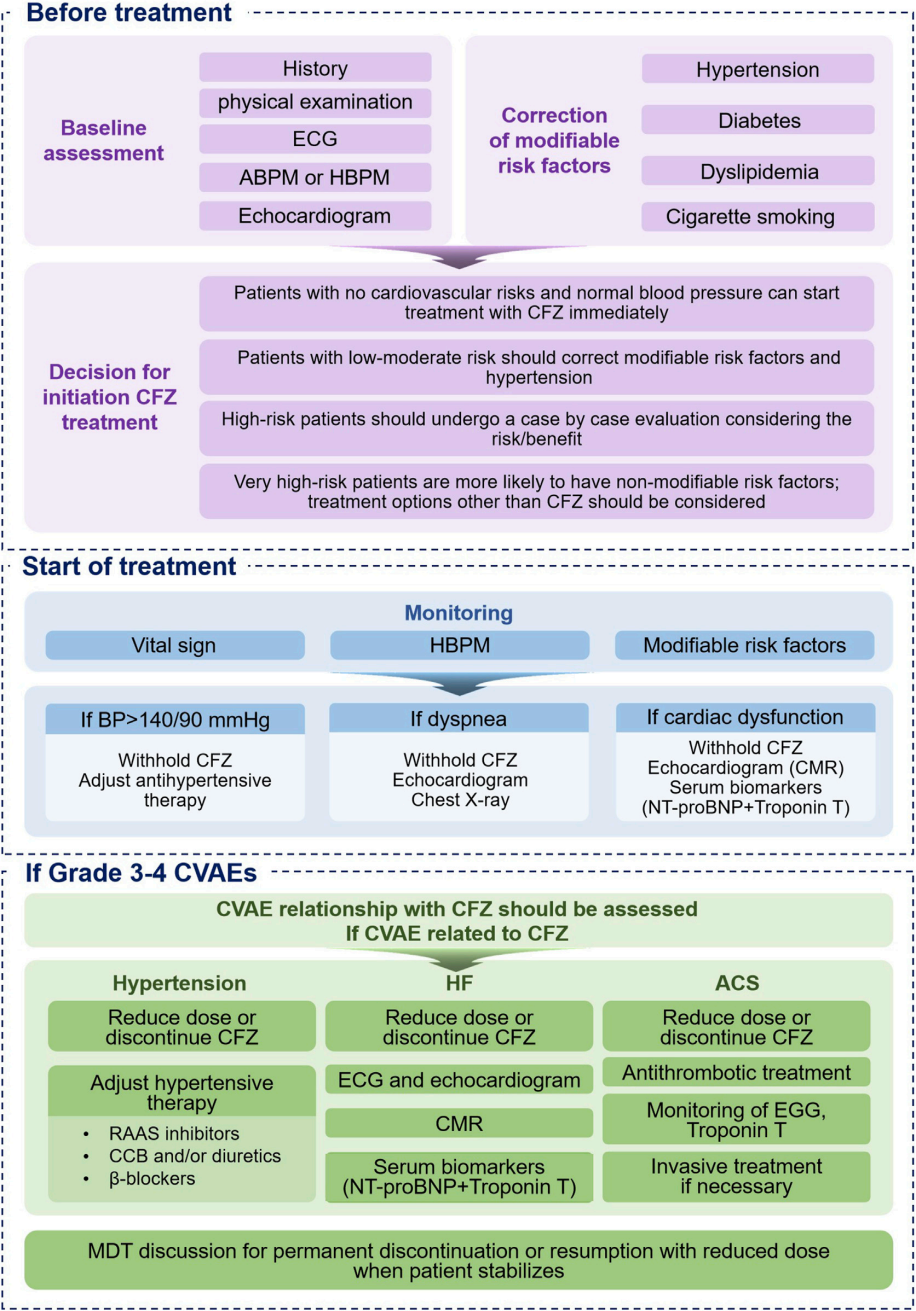
Proposed Management of CFZ-Related Cardiotoxicity. ABPM = ambulatory blood pressure monitoring, BP = blood pressure, CCB = calcium channel blocker, CFZ = carfilzomib, CMR = cardiac magnetic resonance, CVAE = cardiovascular adverse event, ECG = electrocardiogram, HBPM = home blood pressure monitoring, MDT = multidisciplinary, RAAS = renin-angiotensin aldosterone system.

The primary goal of the discipline of cardio-oncology is to enable patients with MM to receive the safest and best possible treatment while minimizing treatment-related cardiovascular toxicity throughout the entire treatment course. Before initiating CFZ treatment, the cardiovascular oncology team should systematically identify and address cardiovascular risk factors and pre-existing cardiovascular conditions. Additionally, they should formulate appropriate prevention and monitoring strategies, including the early detection and effective management of CVAEs (Figure 2).

### 6.1 Clinical evaluation to identify risk factors

Patients should be stratified based on their baseline risk of cardiovascular toxicity before initiating CFZ therapy. This stratification should include clinical assessments and ancillary tests (Bringhen et al., 2019; Lyon et al., 2022). Clinical evaluation entails obtaining a comprehensive medical history from patients to identify prior cardiovascular events and risk factors (including hypertension, diabetes, dyslipidemia, obesity, and smoking) and previous exposure to cardiotoxic agents used for cancer treatment. Moreover, clinical evaluation should also consider genetic predispositions and other factors that may not be immediately apparent. The estimation of all cardiovascular risks should be performed by detailed stratification or with the handier Systematic COronary Risk Evaluation (SCORE) model (http://www.heartscore.org) that estimates the risk of death from cardiovascular disease over 10 years (Conroy et al., 2003).

#### 6.1.1 Baseline cardiovascular risk factors

The baseline cardiovascular risk factors and corresponding scores for patients with MM undergoing treatment with CFZ are as follows (Lyon et al., 2020):

Medium-risk factors, assigned a score of 1, include left ventricular hypertrophy (LVPW >1.2 cm), age 65–74 years, hypertension, diabetes mellitus, hyperlipidemia, chronic kidney disease (estimated glomerular filtration rate <60 mL/min/1.73 m^2^), family history of thrombophilia, prior thoracic spine radiotherapy, high-dose dexamethasone >160 mg/month, current smoker or significant smoking history, and obesity (BMI >30 kg/m2).

Medium-risk factors, assigned a score of 2, include borderline LVEF 50%∼54%, history of arrhythmia (atrial fibrillation, atrial flutter, ventricular tachycardia, or ventricular fibrillation), and elevated baseline troponin.

High-risk factors include a history of prior immunomodulatory drug CV toxicity, history of baseline LVEF <50%, elevated baseline BNP or NT-proBNP, age ≥75 years, and prior anthracycline exposure.

Very high-risk factors include history of heart failure or cardiomyopathy, history of prior PI cardiotoxicity, history of venous thrombosis (DVT or PE), history of cardiac amyloidosis, and history of arterial vascular disease.

#### 6.1.2 Risk stratification

The cardiovascular risk stratification for patients with MM treated with CFZ was as follows:

Low-risk patients had no risk factors or only one medium-risk factor; medium-risk patients had medium-risk factors, with a total score of 2–4; high-risk patients had medium-risk factors or any risk factors with a total score of ≥5; very high-risk patients were identified by the presence of any very high-risk factors (Bringhen et al., 2019; Lyon et al., 2020).

### 6.2 Essential physical examinations

Regular physical evaluations constitute a fundamental component of cardiovascular surveillance, enabling the early detection of preclinical cardiac dysfunction that may precede measurable abnormalities on conventional diagnostic modalities. Essential physical examinations should be conducted, including blood pressure determination and cardiac examinations.

Hypertension represents a potent and modifiable risk factor for cardiac dysfunction onset and should be assessed before starting treatment (Polonsky and DeCara, 2019). For instance, a sudden drop in blood pressure or tachycardia may signal arrhythmic events, while persistent hypertension may be indicative of worsening cardiovascular stress. Home blood pressure monitoring (HBPM) and ambulatory blood pressure monitoring (ABPM) are recommended for blood pressure monitoring (Bringhen et al., 2019). Due to an absence of controlled studies, definitive recommendation for the use of antihypertensive drugs in this scenario is not possible. The most frequently prescribed antihypertensive medications include RAAS inhibitors (angiotensin-converting enzyme inhibitors and angiotensin receptor blockers), CCB, and β-blockers, as well as diuretics (Kreutz et al., 2024).

Clinicians should prioritize an assessment of peripheral edema, a cardinal manifestation of venous pressure elevation indicative of cardiac decompensation, together with monitoring symptoms of dyspnea, angina-equivalent discomfort, or exertional fatigue suggestive of myocardial insufficiency (Bringhen et al., 2019; Lyon et al., 2022).

### 6.3 Cardiac biomarkers

Assessments of cardiac biomarkers, including cardiac troponins and natriuretic peptides, are not routinely recommended for the early detection of cardiotoxicity in clinical practice. However, they may serve adjunctive roles in risk stratification and prognostication (Bringhen et al., 2019).

Cardiac troponins (TnI/TnT), which regulate myocardial contractility, are highly sensitive and specific indicators of myocardial injury. Elevated troponin levels often precede detectable cardiac dysfunction and correlate with CVAEs. Despite a lack of standardized guidelines on the optimal timing and uniformity of detection methods, troponin testing remains cost-effective and straightforward to implement, leading to its widespread adoption in clinical settings (Lyon et al., 2022; Christenson et al., 2015).

Natriuretic peptides (e.g., BNP/NT-proBNP), released in response to myocardial wall stress, are established biomarkers in the diagnosis of heart failure and prognostic stratification. A persistent increase in N-terminal pro-B-type natriuretic peptide (NT-proBNP) levels during chemotherapy is associated with subsequent cardiac dysfunction (Bringhen et al., 2019; Lyon et al., 2022).

### 6.4 Role of imaging

The most frequently utilized parameter for routine cardiotoxicity monitoring is the left ventricular ejection fraction (LVEF) of standard echocardiography. Reductions in LVEF exceeding 10% or 5%, accompanied by symptoms of heart failure, are indicative of cardiotoxicity. Pre-chemotherapy LVEF levels are considered predictive of subsequent cardiotoxicity.

Automated speckle-tracking echocardiography-based assessment of global longitudinal strain (GLS) is increasingly recognized as a method for the identification and measurement of minute disruptions in the systolic function of the left ventricle. GLS quantifies the longitudinal contraction of myocardial tissue and is regarded as an effective indicator for predicting early left ventricular dysfunction and heart failure (Singh et al., 2024).

### 6.5 Determination of when to start and restart CFZ therapy

Patients with no cardiovascular risk and normal blood pressure may begin CFZ treatment immediately, while modifiable risk factors, such as hypertension, should be addressed in those with low to moderate cardiovascular risk prior to initiating CFZ therapy. For high-risk patients, an individualized evaluation is necessary for careful weighing of the risks and benefits. Given that high-risk patients are more likely to have non-modifiable risk factors, alternatives to CFZ treatment should be considered (Takakuwa et al., 2019).

CFZ should be initiated carefully in patients with established heart failure and administered in conjunction with Guideline-Directed Medical Therapy as recommended by their cardiologists. Currently, there is no standardized protocol to determine when to restart CFZ therapy in patients experiencing post-dose cardiac dysfunction. Cardiac complications associated with CFZ are partially reversible. Specifically, the overall improvement rates in the grades of heart failure were 60% in the KRd group versus 37.5% in the Rd group in the ASPIRE trial, 36.8% in the Kd group versus 61.5% in the Vd group in the ENDEAVOR trial, and 50% in the CFZ group versus 14.3% in the best supportive care group in the FOCUS trial, and most patients did not experience long-term sequelae (Chari et al., 2018). The decision to continue or restart CFZ therapy should be made collaboratively by the hematologist and cardiologist by factoring in the patient’s clinical status and carefully weighing the risks and benefits of continuing treatment to minimize cardiac dysfunction (Bringhen et al., 2019; Lyon et al., 2022; Cornell et al., 2019).

### 6.6 Adjuvant therapy to reduce cardiotoxicity during CFZ treatment

There have been advances in research related to exploring adjunctive therapies aimed at reducing cardiac toxicity in patients receiving CFZ treatment. The activation of the sympathetic nervous system and the RAAS may undelie chemotherapy-induced cardiotoxicity. Clinical observations have shown that the combination of angiotensin-converting enzyme inhibitors (ACEi) and β-blockers can mitigate CFZ-induced reductio in the LVEF in patients with heart failure. In addition, metformin (Efentakis et al., 2024; Efentakis et al., 2021), atorvastatin (Efentakis et al., 2024), bupropion (Imam et al., 2024), and zingerone (Alam et al., 2022) have been proposed as interventions to mitigate CFZ-induced CVAEs; however, these drugs are still far from being used in a clinical setting in patient populations due to a lack of adequate clinical evidence.

### 6.7 Multidisciplinary team

A multidisciplinary team consisting of cardiologists and hematologists is crucial to manage patients receiving CFZ. Medications should be chosen carefully based on the patient’s cardiovascular risk stratification, focusing on managing all modifiable cardiovascular risk factors. The decision to discontinue treatment with CFZ, an effective but potentially cardiotoxic drug, in patients with MM who are at high or very high risk of developing cardiovascular disease should be made after discussion with multidisciplinary team that includes the treating oncologist/hematologist and a cardiologist. The effectiveness, safety, and individual cardiovascular risk profile should be carefully evaluated and balanced.

## 7 Conclusion

While CFZ demonstrates efficacy in MM, cardiotoxicity remains a critical concern. Despite ongoing research on CFZ-induced cardiotoxicity, there are significant gaps in the evidence that warrant further investigation. The mechanisms underlying CFZ-induced CVAEs are multifaceted, and the pathophysiology of its associated cardiotoxicity is an area of continued research. Future research endeavors should focus on eclucidation of the compensatory cellular mechanisms associated with myocardial injury, as well as the downstream effector molecules of the UPS. This will enable the identification of potential novel therapeutic targets for the prevention and management of cardiotoxicity. For asymptomatic patients with abnormal indicators (e.g., reduced LVEF, elevated BNP, elevated cardiac troponin), prospective studies are necessary to establish thresholds for pausing or reducing CFZ therapy and to develop stratified management pathways involving the application of dynamic biomarkers (e.g., cardiac troponin). The efficacy of traditional drugs, such as ACE inhibitors/ARBs and β-blockers, when administered with CFZ has not been clarified, and randomized controlled trials are needed to verify their benefit in preventing heart failure. Emerging approaches, such as the use of novel drug delivery systems, hold promise for mitigating toxicity while preserving therapeutic efficacy. Multiomics analyses and prospective trials, particularly those integrating genetic profiling and ethnic differences, will play a pivotal role in the advancement of precision cardiovascular oncology. As evidence from randomized trials accumulates, the findings will require translation into individualized management strategies for patients undergoing CFZ-based therapies.
